# Supplementary material for: Wave storm dynamics and clustering, and their impacts on beach erosion
Source: Camb Prism Coast Futur. 2025 Sep 8;3:e22. doi: 10.1017/cft.2025.10012 (PMC12573695; doi:10.1017/cft.2025.10012)
Supplement: Thilakarathne et al. supplementary material [file S2754720525100127sup001.pdf]

Supplementary materials

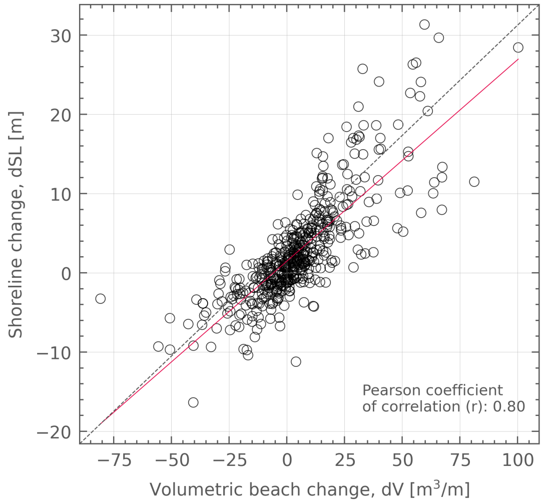

Figure A1. Relationship of volumetric beach erosion, dV, and shoreline erosion, dSL.

Table A1. Characteristics of individual and clustered (shown in parentheses) storms and corresponding geomorphology changes at Hasaki, Japan, in three-year groups. SP: mean storm power ($m^2 \cdot h$), dSL: mean shoreline erosion/accretion (m), dV: mean volumetric beach erosion/accretion (m^3/m).

Period	Number of events		Storm power [$m^2 \cdot h$]		mean shoreline change [m]		mean beach change [m^3/m]	
	Erosional	Accretional	Erosional	Accretional	Erosional	Accretional	Erosional	Accretional
1987–1989	17 (8)	13 (6)	1079.1 (2225.4)	1022.2 (1740.3)	2.69 (10.66)	0.02 (0.08)	8.52 (22.24)	-5.62 (-6.39)
1990–1992	16 (6)	5 (5)	1047.7 (1992.3)	1291.7 (1259.1)	6.02 (10.25)	-3.27 (-1.06)	10.69 (23.24)	-9.67 (-5.53)
1993–1995	22 (2)	6 (3)	1161.3 (773.5)	866.0 (1167.8)	5.38 (4.08)	-1.32 (-5.81)	12.05 (5.13)	-19.16 (-19.83)
1996–1998	19 (8)	15 (3)	859.8 (3284.9)	370.7 (1004.0)	5.09 (12.51)	-0.45 (-0.90)	9.89 (23.65)	-4.84 (-5.72)
1999–2001	27 (10)	18 (1)	972.9 (2023.6)	610.0 (481.9)	4.85 (7.84)	-1.11 (-3.75)	8.76 (15.90)	-4.03 (-5.93)
2002–2004	33 (3)	16 (1)	1148.5 (1975.6)	1400.8 (529.1)	4.96 (12.13)	-1.07 (0.75)	12.47 (29.19)	-10.31 (-7.37)
2005–2007	27 (6)	23 (1)	1714.6 (2342.5)	906.2 (1281.0)	5.94 (11.62)	-0.55 (-0.16)	13.23 (37.71)	-11.60 (-11.19)
2008–2010	0 (0)	1 (0)	— (—)	227.1 (—)	— (—)	-3.06 (—)	— (—)	-24.33 (—)
2011–2013	2 (1)	3 (0)	1923.2 (814.1)	884.0 (—)	12.14 (0.59)	-1.66 (—)	28.58 (14.69)	-9.38 (—)
2014–2016	31 (6)	17 (2)	955.8 (5037.7)	767.8 (1397.4)	3.13 (5.12)	-2.68 (-6.78)	19.75 (26.02)	-19.73 (-43.52)
2017–2019	21 (9)	27 (4)	811.2 (2285.1)	841.9 (1580.0)	4.33 (6.74)	-2.94 (-2.26)	17.41 (22.76)	-15.37 (-15.25)
2020–2022	21 (8)	25 (3)	1352.3 (1915.9)	1455.2 (2194.4)	4.82 (4.42)	-2.79 (-5.05)	21.39 (21.33)	-16.77 (-16.20)
